# Omega 3 Polyunsaturated Fatty Acids Improve Endothelial Dysfunction in Chronic Renal Failure: Role of eNOS Activation and of Oxidative Stress

**DOI:** 10.3390/nu9080895

**Published:** 2017-08-18

**Authors:** Michela Zanetti, Gianluca Gortan Cappellari, Davide Barbetta, Annamaria Semolic, Rocco Barazzoni

**Affiliations:** 1 Department of Medical, Surgical and Health Sciences, University of Trieste, 34149 Trieste, Italy; gigici2@iol.it (G.G.C.); semolic@units.it (A.S.); barazzon@units.it (R.B.); 2 Animal Facility, University of Trieste, 34127 Trieste, Italy; davidebarbetta68@gmail.com

**Keywords:** omega 3 PUFA, endothelial dysfunction, renal disease, nitric oxide, superoxide

## Abstract

Background: Endothelial dysfunction is a key vascular alteration in chronic kidney disease (CKD). Omega 3 (*n*-3) polyunsaturated fatty acids (PUFA) reduce vascular oxidative stress and inflammation. We investigated whether *n*-3 PUFA could reverse endothelial dysfunction in CKD by improving endothelial nitric oxide synthase (eNOS) function and oxidative stress. Methods: 5/6 nephrectomized male Wistar rats (CKD; *n* = 10) and sham operated animals (SHAM; *n* = 10) were treated for 6 weeks with standard diet. An additional group of CKD rats were fed an *n*-3 PUFA enriched diet (CKD + PUFA; *n* = 10). We then measured endothelium-dependent (EDD) and -independent vasodilation, markers of endothelial function and of oxidative stress in thoracic aortas. Results: Compared to SHAM, in CKD aortas EDD and eNOS expression were reduced (*p* < 0.05) and 3-nitrotyrosine levels were increased, while expression of NADPH oxidase subunits NOX4 and p22^phox^ was similar. In-vitro incubation with Tiron failed to reverse endothelial dysfunction in CKD. In CKD + PUFA, EDD improved (*p* < 0.05) compared with CKD rats, while blockade of eNOS by L-NAME worsened EDD. These effects were accompanied by increased (*p* < 0.05) eNOS and reduced (*p* < 0.05) expression of NOX4 and 3-nitrotyrosine levels. Conclusion: Collectively, these findings indicate that *n*-3 PUFA improve endothelial dysfunction by restoring NO bioavailability in CKD.

## 1. Introduction

Omega 3 (*n*-3) polyunsaturated fatty acids (PUFA) cause several beneficial effects on cardiovascular and endothelial function both through direct and indirect mechanisms. Importantly, they are known to increase endothelium-dependent vasodilation by directly enhancing nitric oxide (NO) availability and by reducing oxidative stress and the production of proinflammatory cytokines [1]. In addition, they improve cardiovascular risk factors, such as blood pressure, plasma levels of triglycerides and heart rate [2,3]. On these grounds, *n*-3 PUFA supplementation has been suggested as a therapeutic strategy in primary and secondary prevention of cardiovascular disease.

Endothelial dysfunction has been reported both in chronic kidney disease (CKD) patients and in animal models of chronic renal failure [4,5], and is closely associated with the increased mortality observed in the disease. NO deficiency and oxidative stress play a fundamental role in the pathogenesis of CKD-associated endothelial dysfunction. NO production has been reported to be decreased in CKD [6] due to reduced synthesizing enzyme expression and activity [7,8]. Besides, reduced vascular NO availability in this setting has been described in association with enhanced generation of oxygen free radicals by NADPH oxidases [9,10]. In addition, other factors including increased concentrations of the NOS inhibitor asymmetric dimethylarginine (ADMA) can contribute to endothelial dysfunction during renal failure [11].

In CKD, *n*-3-polyunsaturated fatty acids could therefore potentially improve endothelial dysfunction by inducing anti-oxidant and anti-inflammatory effects and by enhancing NO availability, although specific data are currently not available. Recently, Sikorska et al. [12] and Shoji et al. [13] have investigated *n*-3 PUFA levels in CKD patients and their relationship with cardiovascular mortality. These studies have shown that plasma levels of *n*-3 PUFA are reduced in hemodialysis patients [12] and that low *n*-3 PUFA are independent predictors of cardiovascular disease in this population [13]. However, these results may only be regarded as descriptive and the mechanisms underlying *n*-3 PUFA induced vascular protective effects in CKD still need to be fully clarified. 

Therefore, the aim of this study is to examine the effects of *n*-3 PUFA administration on endothelial dysfunction in an animal model of CKD, and to further investigate the underlying mechanisms.

## 2. Materials and Methods

### 2.1. Animals 

The experimental surgical protocol was approved by the Italian Ministry of Health—Animal Experimentation Authority (DM 274/2013-B 07/11/2013). Thirty 12-week-old Wistar rats (Harlan-Italy, San Pietro al Natisone, Udine, Italy) were used in the experiments. Before surgery, all rats were fed a standard laboratory diet (2018, Harlan, Madison, WI, USA) and were allowed free access to water under a constant light and dark cycle of 12 h. Throughout the study rats were housed in individual cages at the University Animal Facility of Trieste.

### 2.2. In Vivo Uremic Model and Procedures

A 5/6 nephrectomy was chosen as the model of renal disease [14]. Animals were randomly assigned to receive single-step laparotomic 5/6 nephrectomy or sham operation as recently described by the authors of [15]. After anaesthesia (premedication: Dexmedetomidine 0.05 mg/kg IP, anaesthesia: Zoletil 25 mg/kg IP, lidocaine 4 mg/kg local infiltration), median laparotomy, opening of the retro-peritoneum and left kidney isolation, the renal artery was clamped and the inferior and superior poles were resected. Before unclamping, in order to control bleeding, haemostatic absorbable sponges (Spongostan, Ethicon, Sommerville, NJ, USA) and packing were applied. The right kidney was similarly isolated and explanted, after ligature of vessels and of the ureter. After accurate haemostasis check, packing removal and posterior peritoneum continuity reconstruction, the abdominal wall was sutured by mass-layer single absorbable stitches and the skin by single stitches. Volume replacement with saline as well as antalgic therapy were administered subcutaneously for the following days as needed. Sham operated animals underwent the same procedure except for kidney resections and explant. After 10 days from surgery, all animals were free of any complication or treatment.

Immediately after surgery, nephrectomized rats were randomly assigned to standard rat chow diet (*n* = 10; Harlan 2018, 14.2 kJ/g, fat: 6% *w*/*w*, 17% of total Cal; CKD; *n* = 10) or *n*-3 PUFA enriched diet (fat: 6% *w*/*w*, 17% of total Cal; EPA + DHA = 27% total fat). Diet was obtained by substituting soybean oil with highly refined *n*-3 PUFA preparation (EPAX 6000 TG) in Harlan 2018 standard chow, otherwise leaving all other ingredients unmodified (CKD-PUFA; *n* = 10, diet was custom-made by Harlan). EPAX 6000 TG oil (EPA 300mg/g, DHA 200mg/g, other *n*-3 PUFA 100mg/g in triglyceride form, free from other fatty acids) was kindly donated by EPAX, Sandvika, Norway. Sham-operated rats were fed with standard diet (SHAM; *n* = 10).

40 days after surgery, under surgical anaesthesia (Tiobutabarbital 100 mg/kg, tilemine/zolazepam (1:1) 40 mg/kg IP), blood samples were obtained by cardiac puncture followed by aorta collection after accurate dissection and removal of surrounding adipose and connective tissue.

### 2.3. Biochemical Parameters

All reagents were obtained from Sigma (St. Louis, MO, USA) unless stated otherwise. Plasma urea and creatinine concentrations were measured by standard enzymatic-colorimetric assays.

### 2.4. Analysis of Vascular Reactivity

Endothelium-dependent (EDD) and -independent vasodilation in rat aorta was measured ex vivo as previously described [16]. Briefly, immediately after dissection, thoracic aortic rings (2 mm long) were suspended in isolated organ baths organ chambers with gassed (95% O_2_ and 5% CO_2_) modified Krebs-Ringer bicarbonate solution (composition in mmol/L: 118.3 NaCl, 4.7 KCl, 2.5 CaCl_2_, 1.2 MgSO_4_, 1.2 KH_2_PO_4_, 25.0 NaHCO_3_, 0.026 EDTA, 11.1 dextrose, pH 7.4). After equilibration for 1 h at 37 °C, rings were stretched to the optimal point as determined by repeated exposure to cumulative doses of KCl. The maximal contraction of each ring was then assessed by phenylephrine (PHE) 10^−5^ mol/L, followed by washing and re-equilibration. Dose-relaxation curves were finally performed by subsequent cumulative addition of acetylcholine (10^−9^ to 10^−5^ mol/L) or SNP (sodium nitroprussiate; 10^−10^ to 10^−5^ mol/L) to rings in submaximal precontraction state as a result of the addition of 10^−6^ mol/L of PHE. Single dose experiments were similarily performed by addition of acetylcholine (10^−6^ mol/L) to submaximal precontracted rings in presence or absence of the superoxide scavenger Tiron (2 × 10^−4^ mol/L) or of eNOS inhibitor L-NAME (10^−4^ mol/L).

### 2.5. Western Blot

Aortic segments for the measurement of protein levels of eNOS, NOX4, p22^phox^ were isolated at sacrifice and immediately snap frozen in liquid nitrogen for Western Blot analysis [17]. Frozen segments were then homogenized in lysis buffer (50 mmol/L Tris HCl, 0.1 mmol/L EDTA, 0.1 mmol/L EGTA, 0.1% SDS, 0.1% deoxycholate, 1% Igepal, 2 µg/mL leupeptin, 2 µg/mL aprotinin, 1 mmol/L PMSF, 1 µg/mL pepstatin) on ice and centrifuged at 14000 rpm for 10 minutes to remove the insoluble pellet. Protein concentration was assessed by the bicinchoninic acid method (Pierce). 20 µg of protein were separated by SDS/PAGE and semi-dry transferred to 0.2 µm nitrocellulose (Bio-Rad). Blots were blocked with non fat milk (5% *w*/*v*) and incubated with anti-eNOS, anti-NOX4 and anti-p22^phox^ antibodies (1:1000, Transduction Laboratories, Franklin Lakes, NJ, USA, 1:750, Abcam, Cambridge, UK and 1:1000, Abcam, respectively) overnight at 4 °C. After extensive washing and incubation with appropriate horseradish peroxidase-linked secondary antibodies, chemiluminescence detection by x-ray film exposure was performed and the autoradiographs analyzed by densitometry (GS-700, Biorad, Hercules, CA, USA). Equal protein load was confirmed by Ponceau staining and β-actin reprobing.

### 2.6. 3-Nitrotyrosine 

Nitration of protein tyrosine in aortic tissue samples was measured by chemoluminescence-enhanced indirect ELISA, as previously described [18]. 

### 2.7. Statistical Analysis

For each measured variable and each time point, statistical analysis to assess differences among the three groups was performed using unpaired ANOVA followed by post-hoc pairwise t-test comparisons with Benjamini Hochberg correction. Data are presented as mean ± mean standard error unless otherwise specified. *p* < 0.05 was considered significant.

## 3. Results

### 3.1. Animal Characteristics and Phenotype

The 5/6 nephrectomy expectedly resulted in higher (*p* < 0.05) plasma creatinine and urea concentrations. Initial body weight was comparable in all groups (Table 1). Following 5/6 nephrectomy final body weight was lower (*p* < 0.05) in CKD and CKD + PUFA compared with SHAM although total calorie intake measured after nephrectomy was similar in the three groups (Table 1). 

### 3.2. Vascular Reactivity 

**Figure 1:**
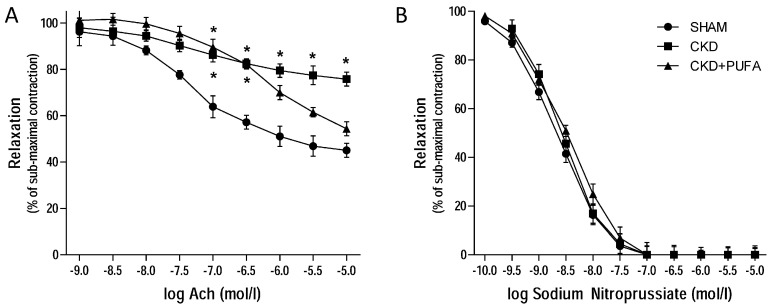
(**A**): Concentration response curves to acetylcholine (ACh) in sham-operated (SHAM), 5/6 nephrectomized rats fed with standard diet (CKD) and fed with *n* 3-PUFA enriched diet (CKD + PUFA). Vascular reactivity studies were performed on segments of thoracic aortas from each group. *n* = 10/group; (**B**): Concentration response curves to Sodium Nitroprussiate in the same groups. *n* = 10/group. Submaximal contraction to phenylephrine (10^−6^ mol/L) was similar among groups. * *p* < 0.05 vs. other groups.

**Figure 2:**
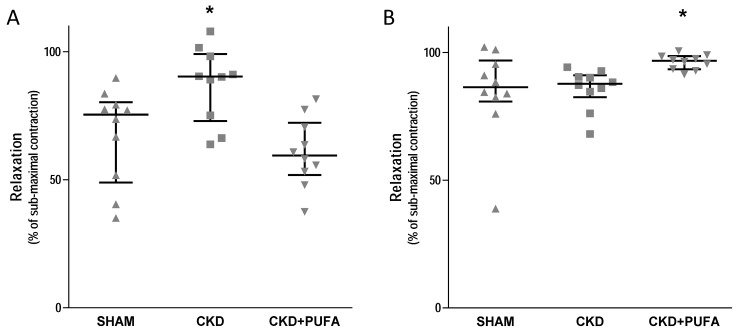
Effect of ex-vivo incubation with the antioxidant Tiron (**A**) and NOS inhibitor L-NAME (**B**) on acetylcholine (10^−6^ mol/L)-induced vasodilation in sham-operated (SHAM), 5/6 nephrectomized rats fed with standard diet (CKD) and fed with n 3-PUFA enriched diet (CKD + PUFA). Vascular reactivity studies were performed on segments of thoracic aortas. Submaximal contraction to phenylephrine (10^−6^ mol/L) was similar among groups. Median and interquartile range. * *p* < 0.05 vs. other groups. *n* = 10/group.

After half-maximal contraction to phenylephrine, endothelial-dependent vasodilation to acetylcholine was impaired (*p* < 0.05) in aortas from CKD rats compared to the control group (Figure 1A). In contrast, endothelium-independent vasodilation to sodium nitroprussiate was similar among groups (Figure 1B). Addition of the antioxidant Tiron did not reverse endothelial dysfunction in aortas from CKD animals fed standard rat chow (Figure 2A). Similarly, in this group NOS inhibition by L-NAME did not modify endothelial dysfunction (Figure 2B). 

Treatment with *n*-3 PUFA resulted in improved (*p* < 0.05) endothelium-dependent vasorelaxation to acetylcholine in CKD rats compared to CKD animals fed with standard diet (Figure 1A). This effect selectively involved the endothelium, since endothelium-independent vasorelaxation to sodium nitroprussiate was similar in the three groups (Figure 1B). Incubation in the presence of Tiron did not further improve endothelium-dependent vasorelaxation to acetylcholine in CKD + PUFA compared to SHAM (Figure 2A). In contrast, addition of L-NAME significantly (*p* < 0.05) attenuated acetylcholine-induced vasodilation, suggesting that improved vasodilation in aortas from CKD animals fed with *n*-3 PUFA is caused by increased nitric oxide synthesis in this group.

### 3.3. Aortic eNOS, NADPH Oxidase Subunits NOX4 and p22^phox^ Expression

**Figure 3:**
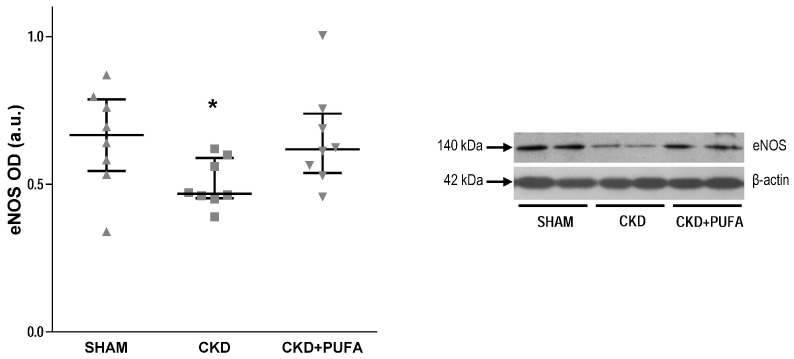
Densitometric analysis of the expression of eNOS protein (140 kDa) in aortas from sham-operated (SHAM), 5/6 nephrectomized rats fed with standard diet (CKD) and fed with *n*-3-PUFA enriched diet (CKD + PUFA), with representative blot. Data represent mean value of 8 animals from each group. In aortas from 5/6 nephrectomized rats fed with standard diet, eNOS expression was lower (* *p* < 0.05) compared to that in vessels from sham-operated and from 5/6 nephrectomized rats fed with *n*-3 PUFA enriched diet. OD: Optical density; a.u.: Arbitrary units. Median and interquartile range.

**Figure 4:**
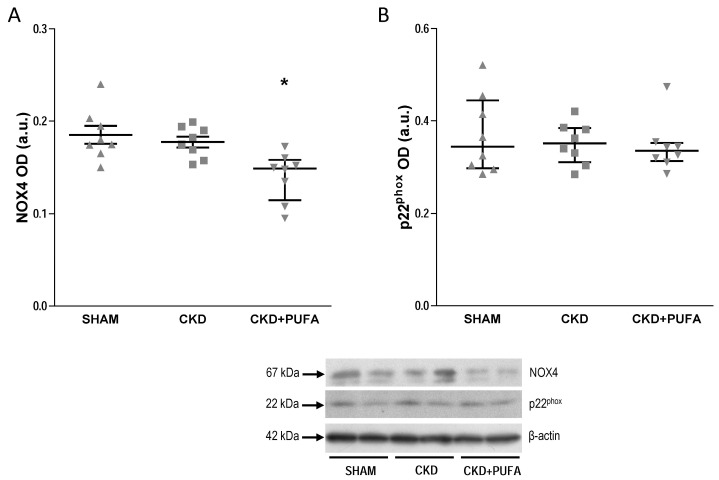
Densitometric analysis of the expression of NAPH oxidase subunit NOX4 (**A**) and p22^phox^ (**B**) in aortas from sham-operated (SHAM), 5/6 nephrectomized rats fed with standard diet (CKD) and fed with *n*-3 PUFA enriched diet (CKD + PUFA). Bottom: Representative western blot. Data represent mean value of 8 animals from each group. In aortas from 5/6 nephrectomized rats fed with *n*-3 PUFA diet, NOX4 expression was lower (* *p* < 0.05) compared to that in vessels from sham-operated and from 5/6 nephrectomized rats fed with standard diet. OD: Optical density; a.u.: Arbitrary units. Median and interquartile range.

CKD rats on standard diet showed a marked (*p* < 0.05) reduction of eNOS expression compared with SHAM (Figure 3). *n*-3 PUFA treatment substantially modified eNOS expression by increasing (*p* < 0.05) its levels to that of SHAM animals (Figure 3). To determine whether changes in endothelial function are also associated with changes in oxidative stress-related enzymes, we assessed the protein levels of the subunits NOX4 and p22^phox^ of the pivotal enzyme NADPH oxidase. Western blot analysis revealed similar expression of NOX4 and p22^phox^ in aortas from SHAM-operated and CKD animals on standard diet (Figure 4). In contrast, in CKD + PUFA, changes in endothelial function and eNOS expression were associated with decreased (*p* < 0.05) tissue NOX4 expression, suggesting a reduction of vascular oxidative stress compared with both SHAM-operated and CKD animals (Figure 4A). Protein expression of the regulatory subunit p22^phox^ was however similar among groups (Figure 4B). 

### 3.4. Aortic 3-Nitrotyrosine Expression

**Figure 5:**
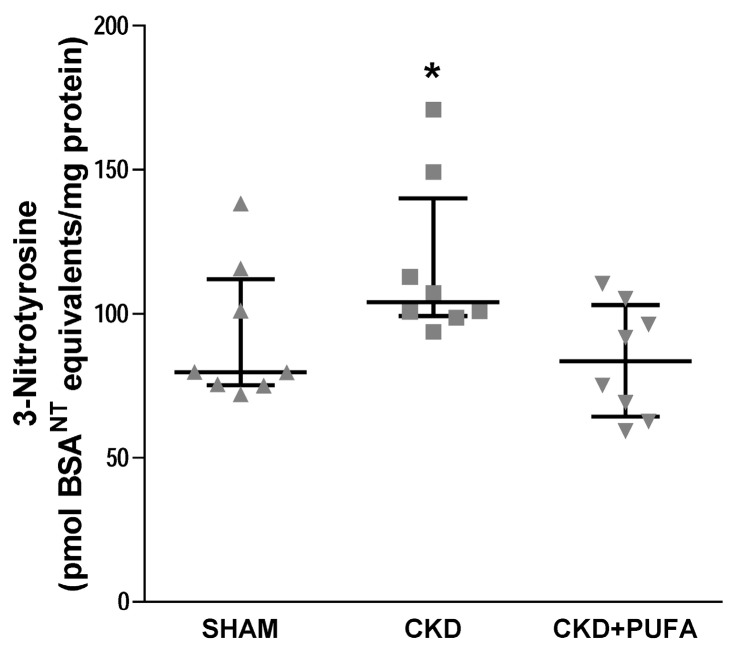
3-Nitrotyrosine content in aortas from sham-operated (SHAM), 5/6 nephrectomized rats fed with standard diet (CKD) and fed with *n*-3 PUFA enriched diet (CKD + PUFA). Data is normalized to total protein content in the homogenized aortic sample and expressed as nitrosylated bovine serum albumin (BSA^NT^) equivalents. In aortas from 5/6 nephrectomized rats fed with standard diet, 3-nitrotyrosine content was increased (* *p* < 0.05) compared with that in vessels from sham-operated and from 5/6 nephrectomized rats fed with *n*-3 PUFA enriched diet. Median and interquartile range. *n* = 8/group.

Aortic expression of 3-nitrotyrosine, a marker of peroxynitrite formation, was higher (*p* < 0.05) in CKD rats on standard diet compared with SHAM (Figure 5). Importantly, *n*-3 PUFA treatment in CKD animals was able to normalize 3-nitrotyrosine levels to a level comparable to that of SHAM animals (Figure 5). 

## 4. Discussion

In this study we found that *n*-3 PUFA supplementation reverses endothelial dysfunction and normalizes reduced eNOS protein expression in aortas from CKD rats. These findings are associated with a substantial reduction of the oxidative damage marker 3-nitrotyrosine, suggesting that in this model decreased oxidative stress may also contribute to the beneficial effect of *n*-3 PUFA on endothelial function.

Human studies have shown that *n*-3 PUFA status in body tissues reflects oral intake [19] and that following incorporation in biological membranes, *n*-3 PUFA specifically activate cardiovascular protective signaling pathways [20], resulting in increased NO production [21], reduced oxidative stress [18,22] and blunted inflammation [23]. Clinical epidemiological studies have demonstrated a significant association between reduced *n*-3 PUFA consumption and the risk of ischemic heart disease [24], suggesting that *n*-3 PUFA replacement may improve clinical outcomes in high-risk populations. In patients undergoing chronic hemodialysis, *n*-3 PUFA reduce all-cause mortality [25], the incidence of myocardial infarction [26] and improves blood pressure [27]. However, their impact on endothelial dysfunction, an early marker of cardiovascular disease is currently unknown in this setting. 

Vascular endothelial dysfunction which occurs during CKD [28] is tightly linked to impaired NO production from eNOS [7,8], as a result of both reduced enzyme expression and activation [5,7]. *n*-3 PUFA increase eNOS expression in the endothelium via several direct and indirect mechanisms, including phosphorylation of AMPK [29] and upregulation of eNOS mRNA [30]; stimulation of SIRT-1 and of heat-shock protein 90 protein expression [31,32]; and finally eNOS translocation from caveolae to the cytosol [33]. 

Consistent with these findings, we found for the first time that reduced eNOS expression and function, as demonstrated by impaired endothelium-dependent vasodilation were reverted by treatment with *n*-3 PUFA in aortas from CKD rats. In addition, incubation of aortas from CKD + PUFA animals in the presence of L-NAME, a NOS inhibitor, completely abolished the beneficial effects of *n*-3 PUFA on endothelial relaxation, indicating that the vasorelaxant response in aortas of CKD animals treated with *n*-3 PUFA is related to increased NO synthesis.

In contrast to endothelium-dependent vasodilation, endothelium-independent vasodilation was not altered in the three experimental groups either under basal conditions or after treatment with *n*-3 PUFA. Interestingly, several studies have suggested that *n*-3 PUFA reduce arterial stiffness and blood pressure [27,34]. Although in this study we did not measure blood pressure, our data collectively suggest that in this experimental setting, *n*-3 PUFA efficacy at the selected dosage is exclusively endothelium-dependent and NO-related. These results are in agreement with animal and human studies performed in CKD showing that *n*-3 PUFA do not influence the mechanical properties of resistance arteries [19,35].

CKD-induced vascular dysfunction can occur either because of impaired eNOS expression and function or because of increased production of reactive oxygen species, which in turn deactivate NO. Oxidative stress is well documented in CKD both in humans and in animal studies [9,36] and can potentially contribute to reduced NO bioavailability and endothelial dysfunction. Interestingly, Hasdan et al. showed that in 5/6 nephrectomized rats, increased oxidative stress occurs as early as 3 days after surgery with normal levels being however restored after 10 days [9]. In accordance with these findings, in ex vivo experiments without the presence of circulating uremic toxins, we found that six weeks after nephrectomy impaired endothelial function in aortas from CKD was not prevented by the antioxidant Tiron, indicating that at this time point of the disease, oxidative stress is not the major contributor to endothelial dysfunction. Accordingly, the expression of the subunits NOX4 and p22^phox^ of NADPH oxidase, a major source of superoxide anion in the vessel wall, was not increased in CKD animals. However, aortic content of 3-nitrotyrosine raised, possibly marking previous peroxynitrite formation in earlier stages of the disease model. 

*n*-3 PUFA are known to blunt NADPH expression and activity [18] and to positively modulate antioxidant potential [37], therefore reducing oxidative stress. In excellent agreement, administration of *n*-3 PUFA to CKD rats lowered the expression of NOX4 but not of the catalytic subunit p22^phox^ compared with aortas of both CKD and SHAM animals. The contribution of reduced NOX4 below physiological levels to improved vasodilation in CKD + PUFA remains to be investigated. Also, aortic expression of 3-nitrotyrosine was normalized following treatment with *n*-3 PUFA. Whether this effect is the result of improved antioxidant potential or of the modulation of oxidative stress pathways alternative to NADPH oxidases cannot be determined from the current data.

Previous studies have shown that in CKD patients *n*-3 PUFA content in plasma and membranes decreases over time and it is positively correlated to fat mass [12]. However, increased fat mass might represent a risk factor for atherosclerosis and cardiovascular events in CKD. Although we did not measure body composition, in our study final body weight was similar in CKD rats independently of the type of diet. Importantly, the normalization of body weight gain in CKD animals treated with *n*-3 PUFA is to be related exclusively to the modification of dietary lipid composition, as total and macronutrient-related calorie intake was not different among groups. 

The *n*-3 PUFA diet contained ~1.6% *w*/*w* of *n*-3 PUFA (60% *w*/*w*) enriched fish oil equal to ~1% energy as EPA and DHA and possibly corresponding to ~2.5 g/day of human equivalent dose [38]. Two metanalyses testing the optimal dose of *n*-3 PUFA (in a range from 0.45 to 4.5 g/day) to correct endothelial dysfunction in humans, did not produce conclusive results mainly because of the heterogeneity of patient populations and of differences in treatment duration [2,39]. Moreover, it must be pointed out that none of the studies included was performed in CKD patients. Identifying dose equivalents between animal and human studies is also controversial and other studies are therefore needed to test the optimum *n*-3 PUFA dose and formulation in the clinical setting of CKD.

In conclusion, we report for the first time that *n*-3 PUFA ameliorate endothelial dysfunction during CKD, and that this finding is associated with increased eNOS expression and function. Further, we found that while expression of NADPH oxidase subunits NOX4 and p22^phox^ is not altered in CKD, increased 3-nitrotyrosine expression is normalized by *n*-3 PUFA. Collectively, these findings suggest a potential therapeutic role for *n*-3 PUFA in CKD-associated endothelial dysfunction, which is likely to be mainly mediated by improved NO bioavailability. 

## Figures and Tables

**Table 1:** Body weight, calorie intake and renal function parameters in sham-operated (SHAM), 5/6 nephrectomized rats fed with standard diet (chronic kidney disease; CKD) and fed with *n* 3-polyunsaturated fatty acids (PUFA) enriched diet (CKD + PUFA). * *p* < 0.05 vs. SHAM

Measurement	SHAM	CKD	CKD + PUFA
N	10	10	10
Initial body weight (g)	359 ± 7	362 ± 4	358 ± 6
Final body weight (g)	452 ± 7	420 ± 10 *	412 ± 15 *
Average daily caloric intake (kcal/day)	63.9 ± 1.9	62.0 ± 2.7	65.2 ± 2.2
Urea (mg/dL)	18.5 ± 3	30.3 ± 1 *	30 ± 1 *
Creatinine (mg/dL)	16.7 ± 1.2	26.8 ± 1.9 *	29.6 ± 1.8 *

## References

[1] Balakumar P., Taneja G. (2012). Fish oil and vascular endothelial protection: Bench to bedside. Free Rad. Biol. Med..

[2] Wang Q., Liang X., Wang L., Lu X., Huang J., Cao J., Li H., Gu D. (2012). Effect of omega-3 fatty acids supplementation on endothelial function: A meta-analysis of randomized controlled trials. Atherosclerosis.

[3] Egert S., Stehle P. (2011). Impact of *n*-3 fatty acids on endothelial function: Results from human interventions studies. Curr. Opin. Clin. Nutr. Metab. Care.

[4] Guajardo I., Ayer A., Johnson AD., Ganz P., Mills C., Donovan C., Scherzer R., Shah S.J., Peralta C.A., Dubin R.F. (2017). Sex differences in vascular dysfunction and cardiovascular outcomes: The cardiac, endothelial function, and arterial stiffness in ESRD (CERES) study. Hemodial. Int..

[5] Chu S., Wang L., Mao X.D., Peng W. (2016). Improvement of Huangqi decoction on endothelial dysfunction in 5/6 nephrectomized rats. Cell. Physiol. Biochem..

[6] Spradley F.T., White J.J., Paulson W.D., Pollock D.M., Pollock J.S. (2013). Differential regulation of nitric oxide synthase function in aorta and tail artery from 5/6 nephrectomized rats. Physiol. Rep..

[7] Nesher N., Frolkis I., Schwartz D., Chernichovski T., Levi S., Pri-Paz Y., Chernin G., Shtabsky A., Ben-Gal Y., Paz Y. (2014). L-arginine improves endothelial function, independently of arginine uptake, in aortas from chronic renal failure female rats. Am. J. Physiol..

[8] Di Pietro N., Giardinelli A., Sirolli V., Riganti C., Di Tomo P., Gazzano E., Di Silvestre S., Panknin C., Cortese-Krott M.M., Csonka C. (2016). Nitric oxide synthetic pathway and cGMP levels are altered in red blood cells from end-stage renal disease patients. Mol. Cell. Biochem..

[9] Hasdan G., Benchetrit S., Rashid G., Green J., Bernheim J., Rathaus M. (2002). Endothelial dysfunction and hypertension in 5/6 nephrectomized rats are mediated by vascular superoxide. Kidney Int..

[10] Nowak K.L., Chonchol M., Ikizler T.A., Farmer-Bailey H., Salas N., Chaudhry R., Wang W., Smits G., Tengesdal I., Dinarello C.A. (2017). IL-1 Inhibition and Vascular Function in CKD. J. Am. Soc. Nephrol..

[11] Chen J., Hamm L.L., Mohler E.R., Hudaihed A., Arora R., Chen C.S., Liu Y., Browne G., Mills K.T., Kleinpeter M.A. (2015). Interrelationship of Multiple Endothelial Dysfunction Biomarkers with Chronic Kidney Disease. PLoS ONE.

[12] Sikorska-Wiśniewska M., Mika A., Śledziński T., Małgorzewicz S., Stepnowski P., Rutkowski B., Chmielewski M. (2017). Disorders of serum omega-3 fatty acid composition in dialyzed patients, and their associations with fat mass. Ren. Fail..

[13] Shoji T., Kakiya R., Hayashi T., Tsujimoto Y., Sonoda M., Shima H., Mori K., Fukumoto S., Tahara H., Shioi A. (2013). Serum *n*-3 and *n*-6 polyunsaturated fatty acid profile as an independent predictor of cardiovascular events in hemodialysis patients. Am. J. Kidney Dis..

[14] Chamberlain R.M., Shirley D.G. (2006). Time course of the renal function response to partial nephrectomy: Measurements in conscious rats. Exp. Physiol..

[15] Gortan Cappellari G., Semolic A., Ruozi G., Vinci P., Guarnieri G., Bortolotti F., Barbetta D., Zanetti M., Giacca M., Barazzoni R. (2017). Unacylated ghrelin normalizes skeletal muscle oxidative stress and prevents muscle catabolism by enhancing tissue mitophagy in experimental chronic kidney disease. FASEB J..

[16] Zanetti M., Gortan Cappellari G., Burekovic I., Barazzoni R., Stebel M., Guarnieri G. (2010). Caloric restriction improves endothelial dysfunction during vascular aging: Effects on nitric oxide synthase isoforms and oxidative stress in rat aorta. Exp. Gerontol..

[17] Gortan Cappellari G., Barazzoni R., Cattin L., Muro A.F., Zanetti M. (2016). Lack of fibronectin extra domain A alternative splicing exacerbates endothelial dysfunction in diabetes. Sci. Rep..

[18] Gortan Cappellari G., Losurdo P., Mazzucco S., Panizon E., Jevnicar M., Macaluso L., Fabris B., Barazzoni R., Biolo G., Carretta R. (2013). Treatment with *n*-3 polyunsaturated fatty acids reverses endothelial dysfunction and oxidative stress in experimental menopause. J. Nutr. Biochem..

[19] Borg M., Svensson M., Povlsen J.V., Schmidt E.B., Aalkjær C., Christensen J.H., Ivarsen P. (2016). Long chain *n*-3 polyunsaturated fatty acids and vascular function in patients with chronic kidney disease and healthy subjects: A cross-sectional and comparative study. BMC Nephrol..

[20] Mason R.P., Jacob R.F., Shrivastava S., Sherratt S.C., Chattopadhyay A. (2016). Eicosapentaenoic acid reduces membrane fluidity, inhibits cholesterol domain formation, and normalizes bilayer width in atherosclerotic-like model membranes. Biochim. Biophys. Acta.

[21] Zhang W., Fu F., Tie R., Liang X., Tian F., Xing W., Li J., Ji L., Xing J., Sun X. (2013). Alpha-linolenic acid intake prevents endothelial dysfunction in high-fat diet-fed streptozotocin rats and underlying mechanisms. Vasa.

[22] Casos K., Zaragozà M., Zarkovic N., Andrisic L., Portero-Otin M., Cacabelod D., Mitjavila M.T. (2010). A fish-oil-rich diet reduces vascular oxidative stress in apoE(-/-) mice. Free Radic. Res..

[23] Hung A.M., Booker C., Ellis C.D., Siew E.D., Graves A.J., Shintani A., Abumrad N.N., Himmerfalb J., Ikizler T.A. (2015). Omega-3 fatty acids inhibit the up-regulation of endothelial chemokines in maintenance hemodialysis patients. Nephrol. Dial. Transplant..

[24] Yagi S., Aihara K., Fukuda D., Takashima A., Hara T., Hotchi J., Ise T., Yamaguchi K., Tobiume T., Iwase T. (2015). Effects of docosahexaenoic acid on the endothelial function in patients with coronary artery disease. J. Atheroscler. Thromb..

[25] Inoue T., Okano K., Tsuruta Y., Tsuruta Y., Tsuchiya K., Akiba T., Nitta K. (2015). Eicosapentaenoic Acid (EPA) Decreases the All-Cause Mortality in Hemodialysis Patients. Intern. Med..

[26] Svensson M., Schmidt E.B., Jorgensen K.A., Christensen J.H., on behalf the OPACH Study Groups (2006). *N*-3 fatty acids as secondary prevention against cardiovascular events in patients who undergo chronic hemodialysis: A randomized, placebo-controlled intervention trial. Clin. J. Am. Soc. Nephrol..

[27] Barden A.E., Burke V., Mas E., Beilin L.J., Puddey I.B., Watts G.F., Irish A.B., Mori T.A. (2015). *n*-3 fatty acids reduce plasma 20-hydroxyeicosatetraenoic acid and blood pressure in patients with chronic kidney disease. J. Hypertens..

[28] Annuk M., Zilmer M., Fellstrom B. (2003). Endothelium-dependent vasodilation and oxidative stress in chronic renal failure: Impact on cardiovascular disease. Kidney Int..

[29] Lee CH., Lee S.D., Ou H.C., Lai S.C., Cheng Y.J. (2014). Eicosapentaenoic acid protects against palmitic acid-induced endothelial dysfunction via activation of the AMPK/eNOS pathway. Int. J. Mol. Sci..

[30] López D., Orta X., Casós K., Sáiz M.P., Puig-Parellada P., Farriol M., Mitjavila M.T. (2004). Upregulation of endothelial nitric oxide synthase in rat aorta after ingestion of fish oil-rich diet. Am. J. Physiol..

[31] Jung S.B., Kwon S.K., Kwon M., Nagar H., Jeon B.H., Irani K., Yoon S.H., Kim C.S. (2013). Docosahexaenoic acid improves vascular function via up-regulation of SIRT1 expression in endothelial cells. Biochem. Biophys. Res. Commun..

[32] Stebbins C.L., Stice J.P., Hart C.M., Mbai F.N., Knowlton A.A. (2008). Effects of dietary decosahexaenoic acid (DHA) on eNOS in human coronary artery endothelial cells. J. Cardiovasc. Pharmacol. Ther..

[33] Omura M., Kobayashi S., Mizukami Y., Mogami K., Todoroki-Ikeda N., Miyake T., Matsuzaki M. (2001). Eicosapentaenoic acid (EPA) induces Ca(2+)-independent activation and translocation of endothelial nitric oxide synthase and endothelium-dependent vasorelaxation. FEBS Lett..

[34] Zanetti M., Grillo A., Losurdo P., Panizon E., Mearelli F., Cattin L., Barazzoni R., Carretta R. (2015). Omega-3 Polyunsaturated Fatty Acids: Structural and Functional Effects on the Vascular Wall. Biomed. Res. Int..

[35] MacLeod D.C., Heagerty A.M., Bund S.J., Lawal T.S., Riemersma R.A. (1994). Effect of dietary polyunsaturated fatty acids on contraction and relaxation of rat femoral resistance arteries. J. Cardiovasc. Pharmacol..

[36] Poulianiti K.P., Kaltsatou A., Mitrou G.I., Jamurtas A.Z., Koutedakis Y., Maridaki M., Stefanidis I., Sakkas G.K., Karatzaferi C. (2016). Systemic redox imbalance in chronic kidney disease: A systematic review. Oxid. Med. Cell. Longev..

[37] Qi X., Qin Z., Tang J., Han P., Xing Q., Wang K., Yu J., Zhou G., Tang M., Wang W. (2017). Omega-3 polyunsaturated fatty acids ameliorates testicular ischemia-reperfusion injury through the induction of Nrf2 and inhibition of NF-κB in rats. Exp. Mol. Pathol..

[38] Slee E.L., McLennan P.L., Owen A.J., Theiss M.L. (2010). Low dietary fish-oil threshold for myocardial membrane *n*-3 PUFA enrichment independent of *n*-6 PUFA intake in rats. J. Lipid Res..

[39] Xin W., Wei W., Li X. (2012). Effect of fish oil supplementation on fasting vascular endothelial function in humans: A meta-analysis of randomized controlled trials. PLoS ONE.

